# Cultivation, nutritional value, bioactive compounds of morels, and their health benefits: A systematic review

**DOI:** 10.3389/fnut.2023.1159029

**Published:** 2023-03-17

**Authors:** Yitong Li, Hongyu Chen, Xi Zhang

**Affiliations:** ^1^Bannerbio Nutraceuticals Inc., Shenzhen, China; ^2^National Engineering Research Center of Edible Fungi, Key Laboratory of Applied Mycological Resources and Utilization of Ministry of Agriculture, Institute of Edible Fungi, Shanghai Academy of Agricultural Sciences, Shanghai, China

**Keywords:** morels, bioactive compounds, health benefits, mycelia, nutritional value

## Abstract

Morels are valuable mushrooms being used as foods and medical substances for a long history. The commonly cultivated morel species include *M. eximia, M. importuna*, and *M*. sextelata in China, *M. conica* and *M. esculenta* in the US. Morels' nutritional profile mainly consists of carbohydrates, proteins, fatty acids, vitamins, minerals, and organic acids, which are also responsible for its complex sensory attributes and health benefits. The bioactive compounds in morels including polysaccharides, phenolics, tocopherols, and ergosterols contribute to the anti-oxidative abilities, anti-inflammation, immunoprotection, gut health preservation, and anti-cancer abilities. This review depicted on the cultivation of morels, major bioactive compounds of different morel species both from fruit bodies and mycelia, and their health benefits to provide a comprehensive understanding of morels and support the future research and applications of morels as high-value functional food sources.

## 1. Introduction

Morels (*Morchella* spp.) are highly valued culinary fungal species with their desirable flavor, umami taste and unique texture. They have a characteristic shape, with a spongy, honeycomb-like cap that is attached to a stem. With a hollow interior and a spongy texture, they are described as having a unique savory and “meaty” flavor. They are usually consumed fresh or processed as a flavoring agent. In addition to the prized sensory properties, morels are reported to show rich in proteins, fibers, vitamins and minerals, and low in carbohydrates and fats (1). Particularly, morels contain higher amounts of potassium, zinc, and selenium than many other common mushrooms (2). Besides their high nutritional value, the bioactive compounds of morels, including polysaccharides, phenolics, tocopherols, and ergosterols, also contribute to their health benefits and make them a potential functional food in nutraceuticals and pharmaceuticals. The health benefits of morels include supporting immune functions, providing antioxidant protection, preventing cardiovascular diseases (CVD), and maintaining digestive heath (3).

Morels distribute across Northern hemisphere, and are harvested in spring and summer (4). Wild morels were mainly found in China, Pakistan, India, Turkey, and North America. Like other ascomycetes, morels follow a life cycle as ascocarp, mycelium, sclerotia, knots or pinhead, primordia, and fruiting body in general (5). Cultivation of morels was very limited because of the strict request of environmental temperature, humidity, illumination, pH, and microbiome dynamics (6, 7). Thus, morels were mainly collected in wild for centuries. While after Ower et al. developed outdoor farming of morels in 1980s (8), commercial cultivation of various morel species became available and the production is rising (6). For example, the annual export of dried morels in China increased from 181,000 kg to 900,000 kg at an average price of $ 160 USD/kg over the past 5 years (3). Due to such prominent economic value and expected growing demand, research about morels involving distribution, cytology of life cycle, artificial cultivation, functionality, and product development et al. draws much attention in recent years. In this review, we provided a comprehensive introduction to the life cycle, cultivation, nutrition value and bioactive compounds of the genus *Morchella*, and discussed their health-promoting effects. This review would facilitate the understanding of morels and provide evidence for the development of morel cultivation and their applications in food and pharmaceutical industries.

## 2. Life cycle and cultivation of morels

**Figure 1:**
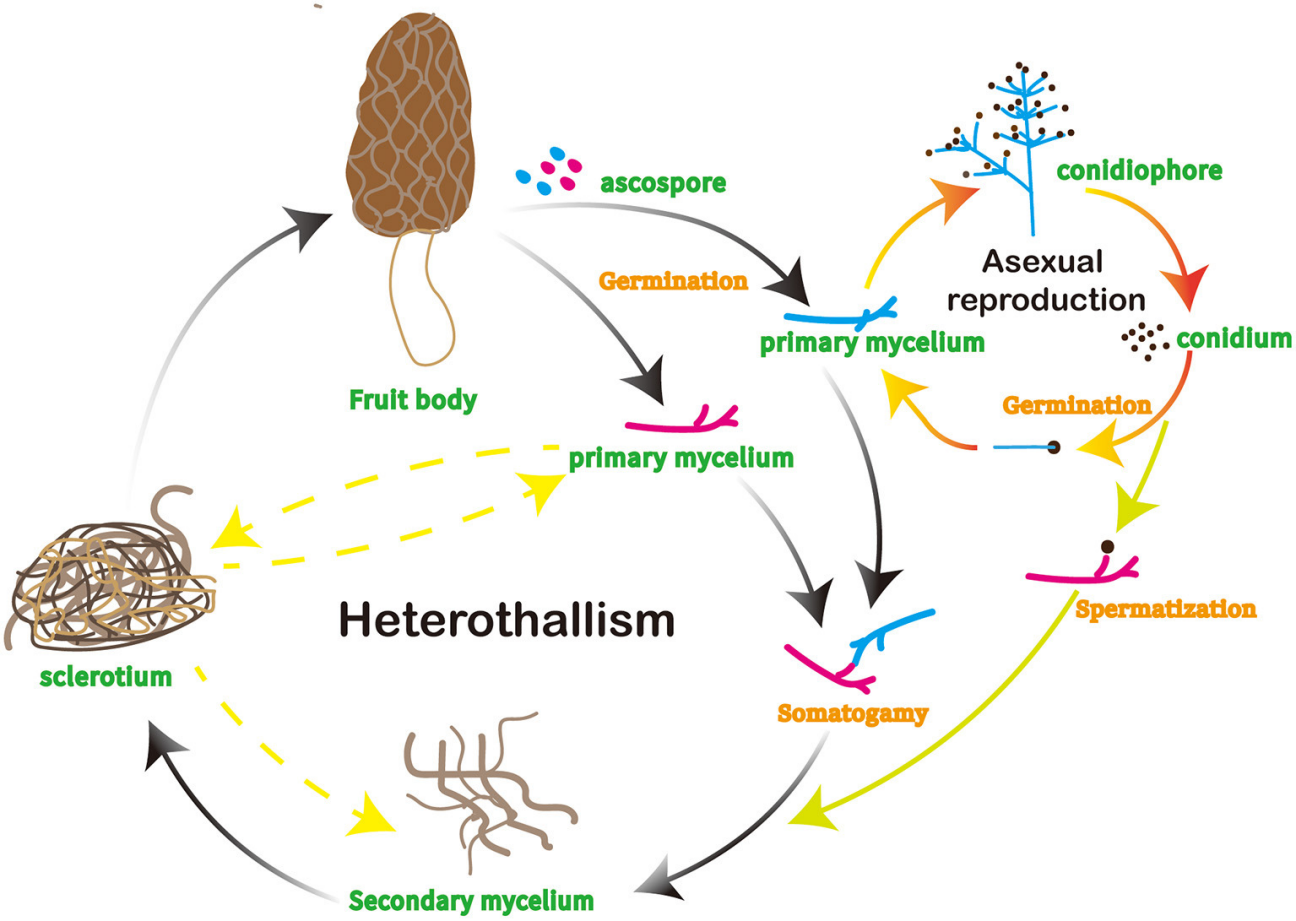
Life cycle of morels.

The life cycle of morels initiates with the dispersal of the mushroom spores into the environment, followed by conidia generation and sclerotia formation (Figure 1) (9). The spores will germinate and form a new mycelium, which is the vegetative part of the fungus and consists of a network of hyphae. Sclerotia-forming hyphae are specialized cells that the fungus creates which start the process of sclerotia production in morels that can tolerate harsh environments (10). The hyphae differentiate and form the sclerotia as the fungus develops and spreads. As the fungus develops and expands, the hyphae that form sclerotia differentiate and produce the fruiting body of the mushroom, comprising a cap and a stem with ridges and valleys (5).

Once the mushroom attains full size, it releases spores into the environment, facilitating reproduction and spread to new areas. Morels also reproduce *via* conidia, which are a type of asexual reproductive structure produced on the surface of the fruiting body (5). Morels typically appear in spring and early summer, marking the beginning of the growing season.

In the wild, the fruiting season of morels only lasts several weeks (9). There is also a concern about the contamination of pesticide residues and heavy metal accumulation in wild morels. The consumption of excessive heavy metal in mushrooms has been associated with growth retardation, impaired immune responses, and malnutrition especially in children (11). To increase the yield and reduce the potential heavy metal and pesticide contamination, the industry of artificial cultivation of morels has been rapidly growing in recent decades. Ower made a breakthrough on the development of the indoor cultivated morels by inoculating the morel's sclerotia and providing exogenous nourishment (8). The morel being first successfully cultivated was published as *Morchella esculenta* and now being described as *Morchella rufobrunnea* (6). Ower's pioneering work laid the foundation for the development of cultivation and commercialization of morels. However, indoor morel production was abandoned in 2008 by the United States due to issues with lower output and bacterial contamination (9).

Many efforts have been made by scientists in China to improve the indoor cultivation of morels. Since 2012, China has successfully implemented the technology of field soil culture for the commercial production of morels (12). Scale morel field farming started with 200 ha and swiftly increased to more than 2,600 ha in 2016, and the number reaches 10,000 in 2018 (13). The highest yield reported reached 15,000 kg/ha, though the yields of morels are usually unstable due to genetic instability and complex reproductive conditions (13). China has been considered as the diversity center of morels due to the genetic variations and floristic diversity (14, 15).

Considering the difficulty of large-scale cultivation of morels and unstable yield of morel fruiting body, the cultivation of its mycelia has been an alternative approach that drawn much attention recently (16). Morel mycelia are the component of the fungus that grows underground and is made up of hyphae, which are interwoven threadlike filaments. It is responsible for consuming nutrients and procreating to create fruiting bodies. The fermentation of morel mycelia dated back to 1960s (17). It was reported that mycelia of morels contained similar or higher amounts of nutrients as its fruiting body. According to Li et al., the protein and unsaturated fat content of *M. esculenta* mycelia was higher than its fruiting body, meaning the mycelia might be a potential high-value protein supplement and a stronger antioxidant compared with the fruiting bodies (17). However, the comparison between the fruiting bodies and mycelia in terms of their health benefits still require scientific evidence.

Despite its well-accepted commercial value, large-scare artificial morel cultivation remains a challenge because of its complicated life cycle and lack of knowledge on the optimal conditions for its fruit body formation (3, 9).

## 3. Cultivated morel species

*Morchella* has been shown to have at least 72 species and 352 isolates including subspecies, the majority of which are provincial and have significant levels of endemism on continents in the Northern Hemisphere (14, 18, 19). They generally distribute in Asia, North America, and Europe. Phylogenetic analysis revealed 30 morel species in China, 22 in the Europe and 19 in the North America (15). Most morel species appear to exhibit continental endemism and provincialism, according to the results of the molecular phylogenetic research, which has made it much easier to reconstruct their historical biogeography (4, 15). The large-scale cultivation of morels has been a rapidly growing market due to their high commercial value and short life cycle (13). After the first successful indoor cultivation of *M*. *rufobrunnea*, evolving technologies such as exogenous nutrition bags have been developed to enhance the efficiency and diversity of cultivated morel species. The most common morel species under cultivation have been summarized in Table 1. The two most prevalent morel species in the United States are *M. conica* (often referred to as the “conical morel”) and *M. esculenta* (also known as the “yellow morel”). *M. importuna, M. eximia*, and *M. sextelata* are the top widely grown species in China, which are known as “black morels” (9). Among them, *M. importuna* accounted for more than 80% of the cultivation space (15).

**Table 1:** Common morel species under cultivation.

**Names**	**Characteristics**	**Cultivation region**
*Morchella conica*	Black morel, “conica morel”, conical, pointed cap	Eastern United States
*Morchella esculenta*	Yellow morel or the “common morel”	Europe, North America, and Asia,
*Morchella deliciosa*	Yellow more, sweet, nutty flavor	Western United States
*Morchella sextelata*	Black morel, small size and dark, spongy cap	China
*Morchella eximia*	Black morel, small, elongated cap and stem	China
*Morchella importuna*	Black morel, small, elongated cap and stem	China
*Morchella rufobrunnea*	Reddish-brown cap	Western United States
*Morchella tomentosa*	Soft, velvety cap	Western United States

The traditional morphological recognition of morels is found to be more complex than expected and could be misleading due to the high morphological plasticity (20). Black morels, yellow morels, and semi-free capped morels were the three groups that the genus *Morchella* was traditionally categorized into (19). Compared to the traditional classification which based on a single morphological trait such as the color or the shape of the pileus, the current morel taxonomy has shifted toward a polythetic approach involving multiple morphological and genetical traits, allowing a more accurate identification of the morels in different areas (20). In the past decade, the development of sequencing technologies and phylogenetic analysis has enabled a more thorough understanding of fungi species and provided more information on the morels' mating system and genes responsible for fruiting and sexual reproduction (21). There are different genetic approaches to distinguish and classify morel species. DNA barcoding has been a useful tool to identify morels using various nuclear gene makers such as the translation elongation factor 1-α (EF1- α), the large subunit of the nuclear ribosomal RNA (LSU), RNA polymerase II largest subunit (RPB1), etc. (19). Genealogical concordance phylogenetic species recognition (GCPSSR) is another method that has been used by many researchers to classify morels. Based on the DNA fragments EF1- α, LSU, RPB1, and GCPSSR was used to study the evolution of morels in China and Turkey, comprising 30 and 62 species, respectively (15).

According to the NCBI GenBank database, the genome sequencing analysis of *M. conica, M. importuna, M. septimelata, M. eximia*, and *M. sextelata* have recently been reported (18, 21–24). Table 2 overviewed the species morels which have been identified through whole genome sequencing. These genomes information would provide additional useful knowledge on the understanding of morel classifications.

**Table 2:** The morel species identified by whole genome sequencing.

**Names**	**Strain**	**Total genome length**	**Scaffold number**	**Contig number**	**GC content**	**N50**	**Genome coverage**	**Reference**
***M. importuna***	M04M26	48.98 Mb	106	111	47.3%	951,626 bp	298 ×	(24)
	M04M24	51.07 Mb	394	763	47.3%	198,406 bp	-	(24)
***M. conica***	CCBAS932	48.21 Mb	540	2,145	47.2%	52,248 bp	67.8 ×	(18)
***M. sextelata***	-	52.93 Mb	-	59	47.37%	1,569,782 bp		(18)
***M. eximia***	MG90	73.46 Mb	7,793	10,613	46.0%	26,474 bp	57 ×	(18)
***M. snyderi***	CBS 144464	54.77 Mb	-	81	-	15 bp	160.8 ×	(21)
***M. septimelata***	MG91	49.96 Mb	6 525	-	47.40%	-	151.17 ×	(23)

## 4. Nutritional value of morels

The nutritional value of morels is highly valued mainly because they are rich in protein, fiber, necessary vitamins and minerals, and low in calories and fat. As shown in Table 3, the total calories of 100 g morels in dry weight ranged from 355.6–386.5 kcal, with 7.5–35.8 g/100 g protein, 2.3–12.0 g/100 g fat, 36.8–80.5 g/100 g carbohydrate, 6.7–18.2 g/100 g ash, 8.5–44 g/100 g total sugar, and 4.8–28.8 g/100 g crude fiber. The compositions varied among reports because of the species, growth area, fertilization method and other environmental conditions. *M. esculenta, M. conica, M. crassipes, and M. elata* are the top morel species studied in terms of their nutritional value.

**Table 3:** Nutrient composition of morels (based on 100 g dry weight).

	**Content**	**Species**	**Origin**	**Reference**
Calories (kcal/100 g)	358.2	*M. elata*	Croatia	(25)
	355.6	*M. conica*	Portugal	(26)
	386.5		Serbia	
	367.6	*M. esculenta*	Portugal	(27)
	379.8		Serbia	
Protein (g/100 g)	22.8	*M. crassipes*	US	(28)
	25	*M. esculenta*	US	(28)
	26.9	*M. hortensis*	US	(28)
	7.5	*M. conica*	Portugal	(26)
	10.1		Serbia	
	24.5	*M. conica*	India	(29)
	35	*M. conica*	Nepal	(30)
	35.8	*M. elata*	Croatia	(25)
Fat (g/100 g)	3.9	*M. elata*	Croatia	(25)
	2.8	*M. conica*	Portugal	(26)
	2.7		Serbia	
	2.6	*M. esculenta*	Portugal	(27)
	2.3		Serbia	
	7.6	*M. crassipes*	US	(28)
	3.3	*M. esculenta*	US	(28)
	3.1	*M. hortensis*	US	(28)
	12	*M. conica*	Nepal	(30)
Carbohydrate (g/100 g)	75	*M. conica*	Portugal	(26)
	80.5		Serbia	
	74.6	*M. esculenta*	Portugal	(27)
	78.4		Serbia	
	36.8	*M. conica*	Nepal	(30)
Ash (g/100 g)	9	*M. elata*	Croatia	(25)
	14.6	*M. conica*	Portugal	(26)
	6.7	Serbia		
	11.3	*M. esculenta*	Portugal	(27)
	7.9	Serbia		
	18.2	*M. crassipes*	US	(28)
	17.3	*M. esculenta*	US	(28)
	17.7	*M. hortensis*	US	(28)
	14.7	*M. conica*	India	(29)
	8.3	*M. conica*	Nepal	(30)
Total sugar (g/100 g)	44	*M. elata*	Croatia	(25)
	8.5	*M. conica*	Portugal	(26)
	9.5		Serbia	
	15.7	*M. esculenta*	Portugal	(27)
	6.4		Serbia	
Crude fiber (g/100 g)	4.8	*M. conica*	India	(29)
	28.8	*M. conica*	Nepal	(30)
Iron (mg/100 g)	53.1	*M. conica*	India	(29)
	25.4	*M. rotunda*	Turkey	(2)
	47.6	*M. crassipes*		
	30.4	*M. esculenta*		
	9.6	*M. deliciosa*		
	7.2	*M. elata*		
	33.6	*M. conica*		
	59.4	*M. angusticeps*		
Zinc (mg/100 g)	2.2	*M. conica*	India	(29)
	7.6	*M. rotunda*	Turkey	(2)
	8.9	*M. crassipes*		
	15.3	*M. esculenta*		
	9.4	*M. deliciosa*		
	12.1	*M. elata*		
	12.6	*M. conica*		
	11.1	*M. angusticeps*		
Copper (mg/100 g)	3	*M. conica*	India	(29)
	2.6	*M. rotunda*	Turkey	(2)
	4.5	*M. crassipes*		
	2.2	*M. esculenta*		
	1.9	*M. deliciosa*		
	3.8	*M. elata*		
	1.2	*M. conica*		
	1.1	*M. angusticeps*		
Magnesium (mg/100 g)	5.6	*M. conica*	India	(29)
	118.4	*M. rotunda*	Turkey	(2)
	149	*M. crassipes*		
	127.2	*M. esculenta*		
	97.4	*M. deliciosa*		
	138.6	*M. elata*		
	169	*M. conica*		
	166.2	*M. angusticeps*		
Calcium (mg/100 g)	248	*M. rotunda*	Turkey	(2)
	356	*M. crassipes*		
	234	*M. esculenta*		
	74.2	*M. deliciosa*		
	135.4	*M. elata*		
	140.4	*M. conica*		
	518	*M. angusticeps*		
Manganese (mg/100 g)	3.5	*M. rotunda*	Turkey	(2)
	3.8	*M. crassipes*		
	2.3	*M. esculenta*		
	1.8	*M. deliciosa*		
	1.4	*M. elata*		
	2.5	*M. conica*		
	4.6	*M. angusticeps*		
Thiamin (Vitamin B1) (mg/100 g)	0.52	*M. hortensis*	US	(31)
Riboflavin (Vitamin B2) (mg/100 g)	1.3	*M. hortensis*	US	(31)
Vitamin C (mg/100 g)	100	*M. conica*	India	(29)
Vitamin D2(mg/100 g)	1.3-7.2	*M. esculenta*	Turkey	(32)
Total phenolic content (mg GAEs/g)	12.3	*M. conica*	India	(29)
	17	*M. rotunda*	Turkey	(2)
	18.6	*M. crassipes*		
	21.3	*M. esculenta*		
	12.4	*M. deliciosa*		
	15.4	*M. elata*		
	25.4	*M. conica*		
	16.6	*M. angusticeps*		
	135.8	*M. deliciosa*	Turkey	(33)
	282	*M. purpurascens*
Total flavonoid content (mg QEs/g)	0.62	*M. conica*	India	(29)
	0.59	*M. rotunda*	Turkey	(2)
	0.47	*M. crassipes*		
	0.25	*M. esculenta*		
	0.15	*M. deliciosa*		
	0.3	*M. elata*		
	0.24	*M. conica*		
	0.26	*M. angusticeps*		

GAEs, gallic acid equivalents; QEs, quercetin equivalent.

### 4.1. Proteins and amino acids

Among them, *M. conica* contained the highest average protein content which reached 24.5 g/100 g (29). The protein content can be increased to 28.1 g/100 g, and the fiber content could reach 16.2 g/100 g in morels with optimized cultivation material formulas (34). The highest crude fiber content was found in *M. conica* from Nepal which reached 28.8% (30). Interestingly, the crude protein content of fermented mycelia of *M. esculenta* was as high as 39.4%, which is much higher than the average protein level of its fruiting body (17). A possible explanation is that during fermentation, complex carbohydrates can be broken down into simpler sugars, which can then be used by the mycelia to produce more protein. Three major soluble monosaccharides were detected in morels, with glucose (42.3 μg/g) being the highest, next to fructose and galactose (35).

The sensory attributes of morels described by the consumers in triangle sensory test are “umami”, “bitter”, “sour”, “mouth-drying”, and “sweet” (22). The amino acids identified in morels mainly includes alanine, l-5-oxoproline and ornithine. The sweetness mainly comes from soluble carbohydrates like mannitol, glucose, and some free amino acids including the most abundant L-alanine, as well as L-serine and L-threonine (36). The complicated sour and mouth-drying taste of morels is also partially ascribed to their abundant amino acid content (22). For example, γ-aminobutyric acid was identified as the specific compound led to the mouth-drying sensation of morels (37). L-glutamic acid and L-aspartic acid add up to the umami flavor in *M. deliciosa*. The amino acid profile varies a lot among species, with the total content ranging from 7.7–56.9 mg/g (25). Threonine and lysine are the major essential amino acids in most mushrooms including morels.

### 4.2. Fatty acids

The fatty acid profile is composed of oleic acid, palmitoleic acid, linoleic acid, α-linoleic acid, palmitic acid, stearic acid and myristic acid (33). According to the study of 6 common morel species, the total saturated fatty acids (SFAs) ranged from 8.4 to 18.5% of the total fatty acid [Dry Weight (DW)], compared with a total monounsaturated fatty acid (MUFA) of 11.7–65.7% (DW), and a total polyunsaturated fatty acid of 14.8–70.1% (DW). It has been shown that MUFA and PUFA (Ployunsaturated Fatty Acid) are the healthy type of fat which presents anti-oxidative abilities (38). Particularly, PUFA are the precursors of some short-chain fatty acids (SCFAs) which are known to contribute to a wide range of health benefits in fungi including anti-inflammation, anti-cancer, and maintaining gut health. Those two types of fat are generally higher in morels than the unhealthy SFAs, indicating the potential health-promoting effects of morels.

### 4.3. Carbohydrates

Carbohydrates play an important role in providing energy for the formation and growth of morel fruiting bodies (35, 39). It accounts for 36.8–80.5% of the morel dry weight (26, 27, 30). Three major soluble monosaccharides were detected in morels, with glucose (42.3 μg/g) being the highest, next to fructose and galactose (35). The polyols in morels are much more abundant than the monosaccharides, which imparts the natural sweetness of morels (35). Volatile carbohydrates including undecane, dodecane, and pentadecane were found in *M. importuna*, imparting herbal and woody aroma of morels (40). Flavor of morel was enhanced by the soluble monosaccharides and sugar alcohols produced by the hydrolysis of stable carbohydrates such as polysaccharides and starch (40). Besides, hydrolysis of the carbohydrates provides substrate for respiration. Mannitol was the predominant sugar alcohol found in *M. importuna* fruiting body (35).

### 4.4. Minerals and vitamins

Wild mushrooms are known to have good mineral concentrations. Altaf et al. found that *M. conica* contains the highest amount of Mg content (55.5 ppm) and high level of Fe (531 ppm) compared to other three wild mushroom species: *Apioperdon pyriforme, Helvella elastica*, and *Rhizopogon luteolus* (29). The study by Gursoy et al. compared the metal contents of seven morel species including *M. rotunda, M. crassipes, M. esculenta, M. deliciosa, M. elata, M. conica, and M. angusticeps*. The results indicated that the highest Mg content (169.0 mg/100 g) was achieved in *M. conica*, the highest Cu content (4.5 mg/100 g) was found in *M. crassipes*, and the Zn content (15.3 mg/100 g) was maximized in *M.esculenta*. *M.angusticeps* contains the highest Mn (4.6 mg/100 g), Fe (59.4 mg/100 g), and Ca (518 mg/100 g) contents (2). Besides, morels contain considerate amounts of vitamin B, vitamin C and vitamin D contents as listed in Table 3. Vitamin C is a well-known antioxidant, and the vitamins are essential for the development and maintenance of immune functions (41).

### 4.5. Organic acids

Several organic acids including succinic acid, malic acid, citric acid, and fumaric acid were also found in morels (35). Succinic acid, as a good antioxidant, was found to be the most abundant organic acid which helps with the mushrooms in fighting diseases and prolonging preservation. The concentrations of the 4 major organic acids in the fruiting body of *M. importuna* ranked from high to low were: succinic acid (308.4 mg/kg), fumaric acid (68.5 mg/kg), citric acid (54 mg/kg), and malic acid (43 mg/kg). They are also responsible for part of the unique taste of morels (35).

In general, morels are a type of tasty, nutrition-dense fungi that contains considerate amounts of proteins, fibers, organic acids, minerals, vitamins, and a good percentage of PUFAs. The complicated taste of morels comes from a series of components including soluble sugars, free amino acids, and organic acids.

## 5. Bioactive compounds

Bioactive compounds of morels mostly include polysaccharides, phenolics, tocopherols, and ergosterols (42–47). Previous studies demonstrated that morels provided a wide range of health benefits not only due to the nutrient profile, but also the bioactive compounds (26, 32, 48). However, the bioactive compounds in mushrooms like morels have always been underestimated. In recent years, growing attention haven been drawn to the bioactivity levels and the phytochemical compositions in different morel species. The bioactive compounds in various morel species, their compositions and functions were reviewed in this section.

### 5.1. Polysaccharides

Polysaccharides in mushrooms can be categorized into β-glucans, α-glucans, and heteroglycans depending on their differences in the glycosidic bonds (49). The average yield of crude polysaccharide-protein complexes in *M. esculenta* fruiting bodies was around 3%, compared to 1.3% of deproteinized polysaccharides (50). The crude polysaccharide consists of a combination of polysaccharides with different molecular weights that have various compositions and functions. A study showed the water-soluble polysaccharides extracted from *M. esculenta* were majorly connected by β type of glycosidic bond (51). The extract had a molecular weight of 43.6 kDa and consisted of glucose, mannose galactose and arabinose. Additionally, the structural and physiochemical traits of polysaccharides were strongly affected by the extraction methods. A study comparing subcritical water extraction (SWE) and hot water extraction (HWE) showed that SWE significantly improved the yield of *M. sextelata* polysaccharides to 18.09% compared with merely 4.95% by HWE (52). Since a higher temperature degraded the polymers and hydrolyzed the side chains, the molecular weight of polysaccharides isolated by SWE decreased.

Polysaccharides, either in the form of β-glucans or as polysaccharide-protein complexes, are the biologically active component of mushrooms and are principally responsible for their therapeutic bioactivity (53). The polysaccharides of *M. esculenta* with a molecular weight of 81 kDa showed anti-proliferation ability in HT29 colon cancer cells; the polysaccharides from wild morels collected from Qinling mountain in China had a high average molecular weight of 3,974 kDa and presented prebiotic effects that help preserve gut microbiota (54). One hypothesis is that the bioactivity of polysaccharides was positively associated with their molecular weight, while the antioxidant activity would be inversely related with the molecular weight (55). β-glucan is one of the most well-known bioactive polysaccharides in mushrooms which showed numerous health benefits including antioxidative, antimicrobial, immunomodulatory and anti-cancer abilities (56). However, the β-glucan in morels lacks documented evidence. Recently, two novel α-D-glucans called MIPB50-W and MIPB50-S-1 were identified and extracted from *M. importuna* fruiting body (46). These two α-D-glucans significantly improve the phagocytosis of macrophages and the secretion of inflammatory cytokines such as TNF -α, IL-6 and essential signaling molecule nitride oxide (NO), which help with the immunoenhancement in human body. Similarly, the polysaccharides isolated from *M. sextelata* were also found to enhance the production of NO and cytokines in the RAW 264.7 cells (52).

As described above, some health-promoting effects including anti-cancer, gut health protection and immunoregulation are ascribed to polysaccharides (47, 49, 53). Therefore, the polysaccharides in morels can be potential functional ingredients that have wide applications on food, cosmetics, pharmaceutical preparations.

### 5.2. Phenolics

Phenolic compounds have been widely reported to prevent bacterial infection, inflammation, CVD, diabetes, and cancer (2, 57). They are known to provide protection to the fungal cell wall against UV radiation and other environmental stress. Phenolic acids and flavonoids are the two major phenolics in morels. The total phenolics and flavonoids of seven morel species were assessed (2). Among the seven morel species, *M. conica* showed the highest phenolic acids content of 25.4 μg GAEs (Gallic Acid Equivalents)/mg extract, and *M. deliciosa* contained the lowest concentration of 12.4 GAEs/mg extract. The flavonoid contents were much lower than the phenolics in general. The highest flavonoid content 0.6 μg QEs (Quercetin Equivalents)/mg extract was observed in *M. rotunda*. Another study investigated six morel species in Turkey showed the highest total phenolic content of 281.96 mg GAEs/g achieved in *M. purpurascens* (33).

**Figure 2:**
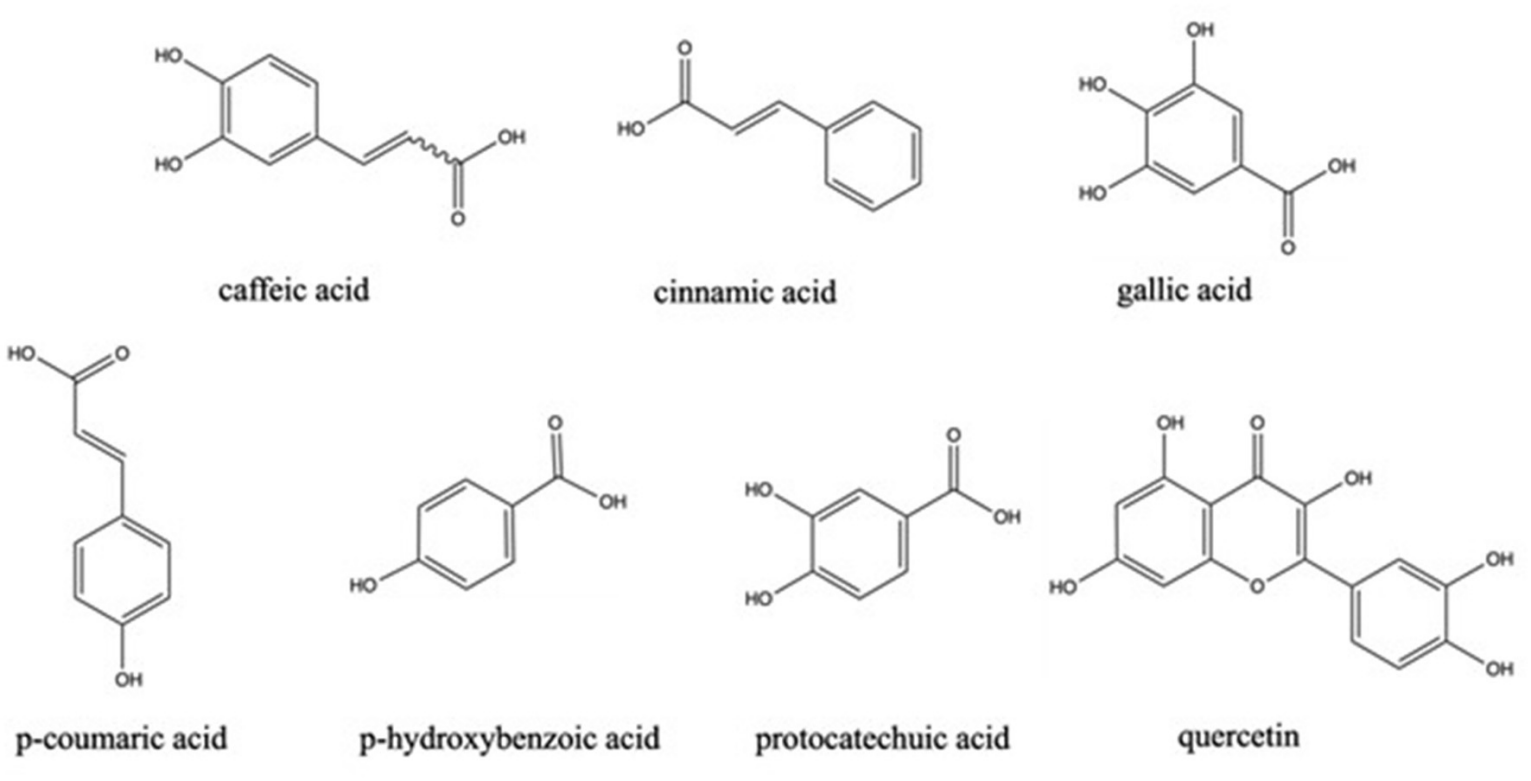
Chemical structure of phenolics in morels.

The phenolic profile of *M. pulchella* was determined by HPLC-MS/MS method (45). The chemical structures of phenolics found in morels are demonstrated in Figure 2. The data indicated that the most abundant phenolic acids in the *M. pulchella* extracts were caffeic acid, which accounted for over 87% of the total phenolics. Caffeic acids could bind to amino acids such as histidine, tyrosine, and serine, inhibiting pancreatic lipase activity, thus maintaining metabolic stability (44). The predominant phenolics found in *M. esculenta* were protocatechuic acid which reached 1,715.2 mg/100 g DW, followed by p-hydroxybenzoic acid (345.8 mg/100 g DW), Quercetin (198.9 mg/100g DW), and gallic acid (78.2 mg/100g DW). In comparison, the *M. esculenta* in India contained the highest quercetin level which was 169.8 mg/kg of the extract, followed by p-Coumaric acid (94.7 mg/kg) (43). A study also compared the bioactive compounds of *M. conica* from different origins (26). It was revealed that gallic acids, p-hydroxybenzoic acids, and cinnamic acid were observed only in Serbian morels, not in Portuguese morels. The highest phenolics in Portuguese morels were the protocatechuic acids which was 20.8 mg/kg DW. They concluded that *M. conica* originated from Serbia contained more phenolics compared with those from Portugal, thus were more beneficial in reducing the risk of chronic diseases. The composition of phenolics varied a lot in different morel species grown in distinct areas and were summarized in Table 4. However, the total phenolic contents found in morels were generally higher than many other wild mushrooms including *Lycoperdon, Ramaria*, and *Clavaria* (43).

**Table 4:** Composition of phenolics in different morel species.

**Species and region**	**Phenolic composition**	**Content**	**Reference**
*M. esculenta* (India)	Quercetin	169.8 mg/kg extract	(43)
	p-Coumaric	94.7 mg/kg extract	
	Rutin	80.0 mg/kg extract	
	Tocopherol	42.6 mg/kg extract	
	Catechol	40.1 mg/kg extract	
	Hyperoside	20.3 mg/kg extract	
	Ellagic acid	13.5 mg/kg extract	
	Cinnamic acid	7.0 mg/kg extract	
*M. esculenta* (Turkey)	Protocatechuic acid	1,715.2 mg/100 g DW	(39)
	p-Hydroxybenzoic acid	345.8 mg/100 g DW	
	Quercetin	198.9 mg/100 g DW	
	Gallic acid	78.2 mg/100 g DW	
	Chlorogenic acid	17.3 mg/100 g DW	
	Epicatechin	12.9 mg/100 g DW	
	Ferulic acid	7.5 mg/100 g DW	
	p-Coumaric acid	0.53 mg/100 g DW	
*M. pulchella* (Turkey)	Caffeic acid	16.2–22.7 mg/g extract	(45)
*M. conica* (Portugal)	Protocatechuic acid	20.8 mg/kg DW	(26)
	p-Coumaric acid	2.5 mg/kg DW
*M. conica* (Serbia)	p-Hydroxybenzoic acid	55.2 mg/kg DW	(26)
	Protocatechuic acid	5.0 mg/kg DW	
	p-Coumaric acid	2.2 mg/kg DW	
	Gallic acid	1.8 mg/kg DW	

### 5.3. Tocopherols

Tocopherols are a group of bioactive compounds that show vitamin E activity and antioxidative abilities. They are hydrophobic antioxidants that are capable of scavenging free radicals and helping with degenerative malfunctions (58). The total tocopherol content in *M.esculenta* fruiting bodies ranged from 14.8 to 121.3 μg/100 g DW (27). The tocopherol level in *M. esculenta* was found to be higher than that of *M. conica*. Besides, the concentrations of tocopherols also varied a lot among regions. The *M. esculenta* from Serbia had a much higher tocopherol concentration than those from Portugal. α-, γ-, and δ- tocopherols are three forms of tocopherols existed in morels. The concentrations of the three forms of tocopherols ranged from a descending order are: δ- tocopherols (48.9–98.6 μg/100 g DW), γ- tocopherols (12.4–20.3 μg/100 g DW), and α-tocopherol (2.4 μg/100 g DW) (53). The study by Mau et al. also showed that the tocopherol content of *M. esculenta* from Taiwan had a higher α- tocopherol content of 0.07 mg/g, followed by γ-tocopherol and δ-tocopherol, with concentrations of 0.06 and 0.04 mg/g, respectively (58).

### 5.4. Ergosterols

Ergosterols are important precursors of vitamin D2 and are the main sterols found in morels. Ergosterol peroxide, the steroidal derivative, widely existed in many edible mushrooms, and provided biological activities (59). Ergosterol peroxide has anti-inflammatory and antimicrobial activities. It's an intermediate in the enzymatic oxidation or the cleavage of reactive oxygen species (ROS), and can be converted back to ergosterol. The content of ergosterol peroxide in *M. esculenta* was 13.4 mg/100 g DW, which was higher than those of *Laetiporus sulfureus* and *Boletus badius* (59). The derivatives of ergosterols were also identified in the study by Lee et al. (42). These novel derivatives of ergosterols isolated were 1-*O*-octadecanoyl-*sn*-glycerol; (3β,5α,8α,22*E*,24*S*)-5,8-epidioxyergosta-6,9(11),22-trien-3-ol; and (3β,5α,22*E*)-Ergosta-7,22,24(28)-trien-3-ol. They found that the three compounds are the main bioactive constituents that contribute to the pro-apoptotic activities of *M. esculenta* toward the human lung adenocarcinoma cells. Four major sterols- 5-dihydroergosterol, ergosterol peroxide, ergosterol, and cerevisterol, were identified in *M. esculenta* which showed anti-oxidative and anti-inflammation abilities (60). The four sterols all significantly inhibited NF-κB activation with a IC_50_ of 2.0–5.2μM. Among the sterols, 5-dihydroergosterol exhibited the strongest inhibition toward NF-κB and highest antioxidant value in the ROS assay with a IC_50_ of 63.1μg/mL.

Ergosterols were also used as the indicator of mycelial biomass in the study to investigate how *M. esculenta* degraded the starch and enhance the nutrition profile of cornmeal under solid-state fermentation (61). The presence of ergosterols added physiological value to the fermented cornmeal product, supporting that ascomycete *M. esculenta* could be used as a potential functional ingredient in novel food development.

## 6. Health benefits

**Figure 3:**
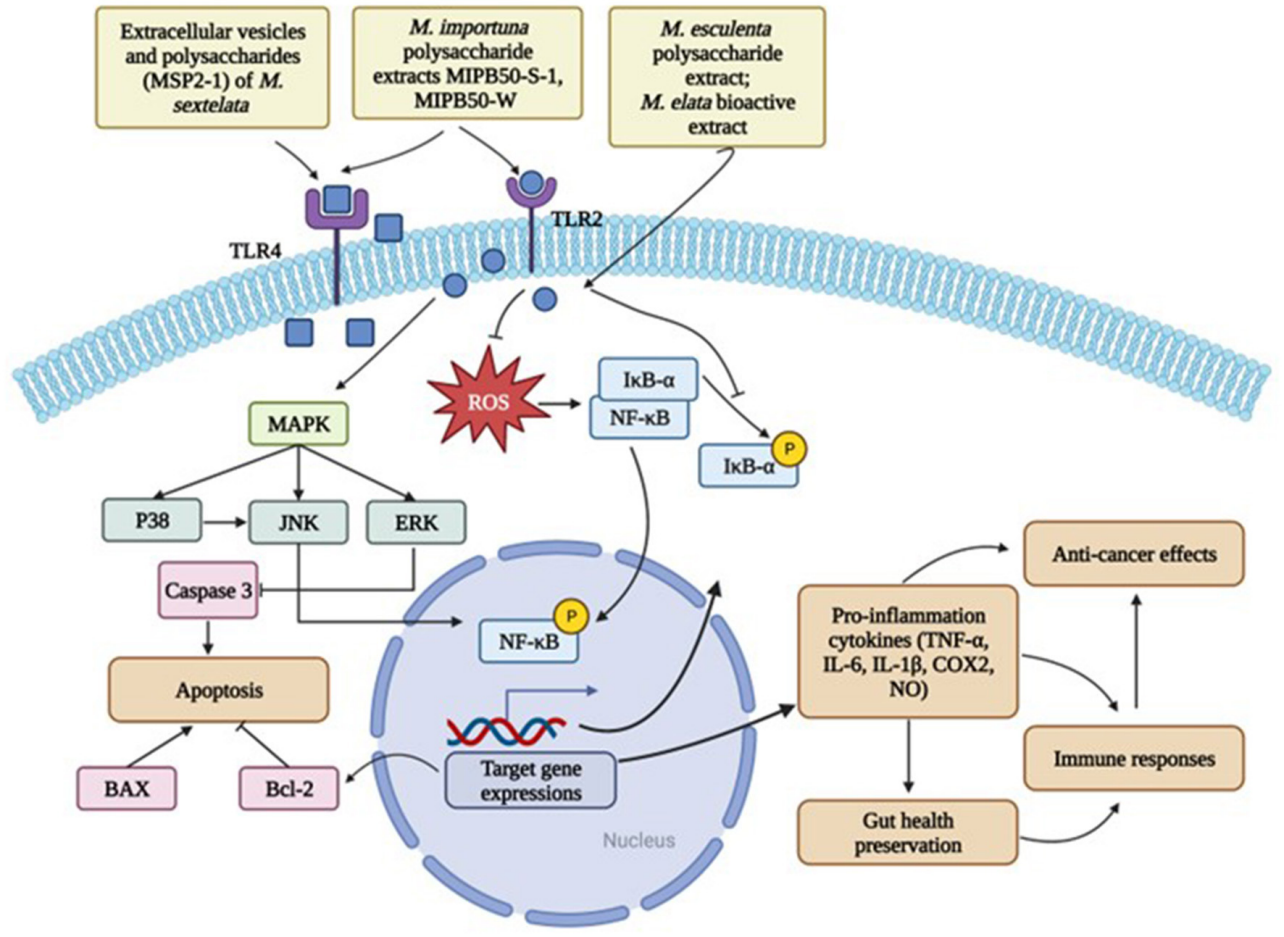
Major cell signaling mechanisms of morel extracts in disease prevention. Created with BioRender.com.

Morels have been used as a traditional medicine in China for thousands of years. The nutrients and bioactive compounds synergistically contribute to the health benefits of morels. Previous research found that morels including their fruiting bodies, mycelia and their extracts showed immunomodulatory, antioxidative, anti-inflammatory, and anti-cancer effects (1). Existing evidence mostly investigated the functions of the two major bioactive compounds: polysaccharides and phenolics in morels. The health benefits of different morel species were summarized in Table 5, and their mechanisms of action was depicted in Figure 3.

**Table 5:** Health benefits of some morel species.

**Samples**	**Extracts/parts**	**Doses**	**Model**	**Effects and mechanisms**	**Health benefits**	**Reference**
*M. elata* fruiting bodies	Bioactive extract	10, 25, and 50 mg	The croton oil induced skin inflammation mice model	Decreasing the thickness of mice skin to an extent from 20 to 75%. NF- κB inhibiting effects	Anti-inflammatory effects	(48)
*M. esculenta* fruiting bodies	Polysaccharide extract (3,947 kDa)	75 mg per kg BW by gavage for 6 days	Non-treated and cyclophosphamide (CP)-treated male Kunming mice	Enhancing the relative abundance of *Bacteroidetes, Ruminococcaceae, Erysipelotrichaceae*, and *Lachnospiraceae* in the mice gut	Immune-protective effects; gut health protective effects	(54)
*M. esculenta* fruiting bodies	Polysaccharides extract	50–400 μg/mL	*In vitro* DPPH, ABTS, and CUPRAC assays	Scavenging free radicals including DPPH, ABTS, and CUPRAC	Anti-oxidative activities	(50)
*M. esculenta* fruiting bodies	Polysaccharides and their derivatives	100, 150, 200, and 400 μg/mL	PM2.5-treated macrophage NR8383 cells	Increasing the ROS release and the production of pro-inflammatory cytokines via the regulation on NF- κB pathway	Anti-inflammatory effects	(62)
*M. esculenta* fruiting bodies	Polysaccharides	75 mg/kg body weight for 8 days	Male Kunming mice	Increasing the beneficial bacterial density and short chain fatty acids in mice	Gut health protective effects	(47)
*M. esculenta* fruiting bodies	Polysaccharides	200 and 400 mg/kg for 12 weeks	Obese BALB/c mice	Recovering the diversity of bacteria, decreasing the abundance of *Firmicutes* and enhancing the abundance of *Bacteroidetes*	Gut health protective effects	(63)
*M. esculenta* fruiting bodies	Polysaccharides	200, 400, and 600 mg/kg	Type 2 diabetic mice model	Improving both *Firmicutes* and *Actinobacteria*, and increasing the intestinal permeability	Gut health protective effects	(64)
*M. esculenta* fruiting bodies	Polysaccharides extract by pulsed electric field (81,835 kda)	200 to 1,000 μg/mL for 24 or 48 h	human colon cancer HT-29 cells	Inhibiting the cell proliferation in a dose and time-dependent	Anti-colon cancer effects	(65)
*M. esculenta* fruiting bodies	Methylene chloride extract	IC_50_ ranged from 2.0–5.2 μM	HT-29 colon cancer cell	Demonstrating high antioxidant activity *via* inhibiting NF-κB	Anti-colon cancer effects	(60)
*M. esculenta* fruiting bodies	Methanolic extract	IC_50_ ranged from 133.1 to 278.0 μM	Human lung adenocarcinoma cell lines A549, H1264, H1299, and Calu-6	The derivatives of octadecanoic acid and ergosterol were the main constituents that responsible for the ability to induce apoptosis in the lung cancer cells.	Anti-lung cancer effects	(42)
*M. esculenta* mycelia	Ethanol extract	0.1, 0.5, and 1% of the extract	*In vitro* DPPH, ABTS, FRAP assays	Scavenging free radicals in a dose-dependent manner	Anti-oxidative activities	(66)
*M. esculenta* mycelia	Ethanol extracts	250, 500, and 1,000 mg/kg	DLA cell line-induced solid tumor and EAC cell line-induced ascites tumor models in mice	Alleviating both acute and chronic inflammation; reducing the weight of the transplanted tumor by 41.1, 61.7, and 76.9% in mice	Anti-inflammatory effects; anti-tumor effects	(67)
*M. importuna* fruiting bodies	Glucan MIPB50-S-1 (444.5 kDa), MIPB50-W (939.2 kDa)	7.81–500 μg/mL	RAW264.7 macrophage cells	Activation of TLR4-NF-κB and MAPK signaling	Immune-protective effects	(46)
*M. importuna* fruiting bodies	Polysaccharides MIPB70-1(20.6kDa)	7.8–500 μg/mL *in vitro*, 50 or 150 mg/kg by gavage *in vivo*	RAW264.7 macrophage cells; BALB/c female mice	Enhancing the phagocytic function and secretion of NO and cytokines *via* the TLR4 signaling; enhancing the anti-tumor activity of doxorubicin (DOX), inhibiting the growth of breast tumor	Immune-protective effects; anti-tumor effects	(68)
*M. importuna* fruiting bodies	Polysaccharides extract	50–400 μg/mL	Mouse neuronal PC12 cells injured by H2O2	Improving the ERK expression and inhibit the p38 and NF-κB expression; depressing caspase-3 and reducing the ratio of Bax-2/Bcl-2 ratio	Anti-oxidative activities	(69)
*M. sextelata* fruiting bodies	polysaccharide MSP-3-1 (23500 kDa)	1.0–5.0 mg/mL	RAW264.7 macrophage cells	Enhancing the cell proliferation, NO production and phagocytosis; antioxidant effects against FPPH and ABTS radicals	Immune-protective effects; anti-oxidative activities	(70)
*M. sextelata* fruiting bodies	Polysaccharide MSP2-1 (371.5 kDa)	25–400 μg/mL	RAW 264.7 macrophage cells	Stimulating the production of NO, cytokines, and promote the proliferation *via* TLR4 receptors	Immune-protective effects	(52)
*M. sextelata* fruiting bodies	Polysaccharides extract (average MW1350kDa)	50 and 100 μg/ml	Mouse neuronal PC12 cells injured by H2O2	Free radical scavenging ability and recovery of injured PC12 cells	Anti-oxidative activities	(55)
*M. sextelata* mycelia	Extracellular vesicles	0.2, 0.9, and 3.5 μg/mL	RAW246.7 macrophage cells	Inhibiting inflammation related factors including TNF-α, IL-6, iNOS and COX-2 via suppressing the MAPK pathway	Anti-inflammatory effects	(71)
*M. conica, M. esculenta, M. delicosa* fruiting bodies	Methanolic and ethanolic extracts	IC_50_ ranged from 0.15–0.66 mg/mL	SW-480 colon cancer cell	Extracts of *M. conica, M. esculenta*, and *M. delicosa* showed anti-proliferative activities with the IC_50_ of 0.41–0.63, 0.15–0.43, and 0.41–0.66 mg/mL, respectively.	Anti-colon cancer effects	(72)
*M. dunalii, M. purpurascens, M. deliciosa, M. mediterraneensis, M. importuna, M. esculenta*	Methanol extract of fruiting bodies	Total phenolic content 135.80–281.96 mg GAE/g dry weight	*In vitro* DPPH, FRAP assays	*M. purpurascens* group behaved best in Scavenging DPPH and FRAP free radicals	Anti-oxidative activities	(33)
*M. rotunda, M. crassipes, M. esculenta, M. deliciosa, M. elata, M. conica, M. angusticeps*	Methanol extract of fruiting bodies	40 μg/mL	*In vitro* DPPH, ABTS assays	The radical scavenging ability was maximized in *M. conica*, followed by *M. crassipes* and *M. esculenta*	Anti-oxidative activities	(2)
*M. vulgaris, M. esculanta* fruiting bodies	Ethanol extracts	50, 100, and 150 μg/mL	*In vitro* DPPH, superoxide anion radical assays	Scavenging the free radicals, superoxide anion radicals and hydrogen peroxide	Anti-oxidative activities	(73)

### 6.1. Supporting immune functions

The immunoregulative effects of morels have been widely studied in recent years. It has been shown that polysaccharides are the primary components responsible for the protective effects of morels on the immune system (49–52, 74).

Glucans, as an important category of polysaccharides, greatly contribute to the immunomodulatory ability of morels. It has been widely reported that β-glucans in mushrooms can stimulate the production of white blood cells, which are important for fighting off infections and diseases (56). However, the evidence of detectable β-glucans in morels lacks documented evidence. Most reports showed the polysaccharides in morels mainly consist of α-glucans (70, 75). Zhang et al. extracted a novel α-glucan, MSP-3-1, from *M. sextelata* and assessed its biological activities (70). MSP-3-1 was with a large molecular weight of 23,500 kDa and basically made of mannose, glucose, and galactose. It possessed immunoprotective abilities by enhancing the cell proliferation, NO production and phagocytosis of the macrophage RAW264.7 cells. The results were confirmed in the study by Li et al. where they found the polysaccharide MSP2-1 extracted from *M. sextelata* also stimulated the production of NO, cytokines, and promote the proliferation of the RAW 264.7 cells via TLR4 receptors. The mechanisms are closely associated with the structure (52). Previous research demonstrated that glucans with glycosidic bonds as → 4)-α-d-Glcp-(1 →, → 3)-β-d-Glcp-(1 → and → 6)-β-d-Glcp-(1 → were more likely to benefit the immune system (46). Interestingly, in the study by Wen et al., the novel glucan MIPB50-S-1 with a molecular weight of 444.5 kDa showed a superior performance on the immunomodulation tests than the MIPB50-W which had a much higher molecular weight (46). This can be explained by the fact that MIPB50-S-1 contained longer side chains such like → 6)-α-d-Glc*p*-(1 →. They also found that both morel glucans significantly enhance the production of TNF-α, IL-6, and NO, which were modulated by the transcription factor NF-κB. The immunoenhancing ability was achieved through the activation of TLR4-NF-κB and MAPK signaling pathways (53).

By using cyclophosphamide-treated mice model, Huo et al. examined the immune-protective effects of the polysaccharide extracted from wild morels (54). The results showed that dietary intake of morel polysaccharides recovered the spleen condition, increasing the white blood cell and lymphocytes counts including CD4+CD8–, CD4–CD8+, and CD4–CD8–CD19+ lymphocytes in the spleen and blood of the mice (54). The effects are possibly associated with their ability to modulate gut microbiota because a balanced gut environment is known to contribute to the immune health of the hosts (76). It was reported that a triple helix was required for the polysaccharides to express immunoactivity which could not be easily achieved in small polymers (55). Therefore, the higher structure of polysaccharides played an important role in affecting their immunoregulative functions. Interestingly, MIPB70-1, the alkaline extraction from *M. importuna* fruiting body with a small molecular weight (20.6 kDa), was also found to exert immunomodulatory effects *in vitro* and *in vivo* (68).

Overall, the mechanisms of action of morels in preserving immune health include the activation of immune cells and responses (46, 68, 70, 74), phagocytosis enhancement (70), and regulation of gut microbiota (55), etc. In details, morel glucans bind to the receptors on immune cells, such as the TLR4 on macrophages (52, 68), thus triggering the activation of the signaling cascade and downstream regulators which in turn leads to the release of cytokines and other molecules that enhance phagocytosis and help limit infections.

### 6.2. Anti-oxidative activities

Excessive ROS are known to damage the cellular organelles and functions, leading to a lot of human diseases. Scavenging of free radicals has been shown as a basic mechanism for the prevention of many chronic diseases including CVD, obesity, and cancer (77). Different species of morels were reported to have antioxidant abilities correlated to their phenolic compounds and polysaccharides (43, 45, 58, 60, 75).

Bioactive compounds extracted from plants have been extensively reported to impose health benefits by stabilizing oxidation processes and reducing the harm to cellular structures (78, 79). The free radical scavenging ability of morel extracts were studied. The ethanol extract of *M. esculenta* mycelia possessed strong antioxidant ability in scavenging superoxide, nitric oxide, 2,2'-diphenyl-1-picrylhydrazyl (DPPH), and 2,2'-azino-bis (3-ethylbenzthiazoline6-sulphonic acid) (ABTS) radicals in a dose-dependent manner (66). The ethanol extracts of *M. vulgaris* and *M. esculanta* fruit bodies in Turkey presented similar properties to scavenge the free radicals, superoxide anion radicals and hydrogen peroxide (74). The study by Vieira et al. showed methanolic extract of *M. conica* from Serbia exhibited a higher reducing power with EC_50_ value of 1.9 units compared to the *M.conica* from Portugal with a EC_50_ value of 1.2 units (26).

Polysaccharides extracted from morels are also considered as important antioxidants. The anti-oxidative activity of the polysaccharides extracted from *M. esculenta* was evaluated by DPPH, ABTS, and CUPRAC assays (50). The *M. Sextelata* polysaccharide MSP-3-1 exhibited scavenging activity against FPPH and ABTS radicals in a dose-dependent manner (70). The impact of γ-irradiation on the anti-oxidative activities of polysaccharides extracted from *M. Sextelata* fruiting body was investigated by Xiong et al. (55). They observed stronger free radical scavenging ability and recovery of injured PC12 cells in irradiation-treated polysaccharides which had a reduced molecular weight. The similar cell model was used in another study by Xiong et al. where they found *M. importuna* polysaccharide suppressed NF-κB pathway activation as well as potential upstream regulatory components including p38 and JNK1/2 (69). Additionally, ERK1/2 expression was elevated and caspase-3 activity was suppressed. All these outcomes resulted in lessened H_2_O_2_-induced oxidative stress damage in PC12 cells (69).

The anti-oxidative abilities of morels also partially attribute to their abundant phenolics. Several studies indicated that morels with higher total phenolic contents showed stronger antioxidative power (33, 43, 45). For instance, the study of six morel species in Turkey showed that *M. purpurascens* group presented highest total phenolic acids behaved best in the DPPH and FRAP assays, indicating it had the strongest reducing potential (33). Strong correlation between the amount of phenolics and the antioxidant characteristic was also observed by Gursoy et al. (2). They found the radical scavenging ability was maximized in *M. conica* which had the most abundant phenolics, followed by *M. crassipes* and *M. esculenta* (2).

Most studies have been focused on the anti-oxidative abilities of morel polysaccharides and phenolics. Tocopherols also contribute to the antioxidative abilities but few scientific work has been focused on them due to comparatively small amounts (80). Phenols, including BHT (butylated hydroxytoluene) and gallates, were reported as effective antioxidants in mushroom fruiting bodies and mycelia (81).

### 6.3. Anti-inflammatory effects

Inflammation is a natural response to injury or infection, but chronic inflammation has also been linked to the development of heart disease and other conditions (82). Some studies have suggested that morels and their mycelia may have anti-inflammatory effects, which could be beneficial for the cardiovascular system (48, 62, 63, 67).

The anti-inflammatory effects of polysaccharides and their derivatives of *M. esculenta* were studied using a PM2.5-treated macrophage NR8383 cell line (62). The extracts, SFMP-1 (sulfated polysaccharide derivatives) and CFMP-1 (carboxymethylated derivatives), exhibited profound anti-inflammation activities against the PM2.5-induced inflammation through significantly ameliorating the production TNF-α and IL-1β in the cells. SFMP-1 reversed the activation NF-κB and downregulated the expressions of iNOS and COX-2 induced by PM2.5 treatment, which indicated that the regulation on NF- κB pathway was the key mechanism for the anti-inflammatory effects of *M. esculenta*.

The extracellular vesicles (EVs) of *M. Sextelata* also demonstrated anti-inflammatory abilities toward the LPS (lipopolysaccharides)-induced inflammation on the RAW246.7 macrophages (71). The inflammation related factors including TNF-α, IL-6, iNOS, and COX-2 were all inhibited by the EVs in a dose-dependent way. The *M. Sextelata* EVs containing a series of lipids, proteins, and micro RNAs, dramatically inhibited the expression of p38 MAPK and the inhibitory effects were even stronger with the addition of 10 mM of the ROS inhibitor NAC (N-acetyl-l-cysteine). That suggested a synergistic effect of NAC and EVs in inhibiting LPS- induced inflammation in the RAW246.7 macrophages. Consistently, acetylated polysaccharides from *Morchella angusticeps* Peck ameliorated inflammation in the LPS-treated RAW246.7 macrophages (83). The same model was used to evaluate the anti-inflammatory ability of the ethyl acetate extract of *M. esculenta* as well (84). In this study, the *M. esculenta* ethyl acetate extract contained 61% flavonoids and showed positive effects on reducing inflammation *via* MAPK signaling pathway.

The anti-inflammatory activities of morels have also been investigated in mice models. It was suggested that the ethanolic extract of *M. esculenta* mycelium alleviated both acute and chronic inflammation when administered with 250 and 500 mg/kg body weight in mice in a dose-dependent manner (67). Even compared with the 10 mg/kg oral administration of the reference drug Diciofenax, 500 mg/kg of *M. esculenta* mycelium extract showed a better performance of anti-inflammation. *M. esculenta* polysaccharides also attenuated high fat diet-induced obesity and chronic inflammation in mice through NF-κB inactivation (63). Similarly, the extracts from the *M. elata* fruiting body demonstrated strong NF-κB inhibiting effects in the croton oil induced inflammation mice model (48). The three doses 10, 25, and 50 mg of the extracts notably decreased the thickness of mice skin induced by croton oil to an extent from 20 to 75%. The results proved that *M. elata* exerted protective effects on skin inflammation.

Both *in vitro* and *in vivo* studies indicated that different parts of various morel species were able to alleviate inflammations caused by distinct stimuli including exposure to fine particles, or administration of LPS and other lipids. Thus, morels and their extracts might be natural anti-inflammatory ingredients in preventing inflammation-related diseases such as rheumatic edema and CVD.

### 6.4. Digestive health protection

The maintenance of balanced gut microbiota is positively associated with the prevention of many chronic diseases including obesity, type 2 diabetes, CVD, and cancer (47, 49). The investigation of morel polysaccharides and gut health has been a popular trend in recent years. Edible morels have been suggested to support digestive health through regulating the gut microbiota due to their rich dietary fiber and bioactive compounds.

Morels contain a considerable amount of fiber, ranged from 4.8 to 28.8% DW. Fibers are widely accepted as the most essential nutrient for gut health (85). In contract to insoluble dietary fiber, soluble dietary fiber is known to readily absorbed and digested by fiber-degrading bacteria in the colon, where it produces a number of functional metabolites including SCFAs (29, 30). The soluble fiber extracted from *M. importuna* enhanced the abundance and diversity of beneficial bacteria such as *Parasutterella, Ruminococcaceae, Faecalibacterium*, and *Lactobacillus*, and increased the production of SCFAs after *in vitro* fecal fermentation (86).

The incorporation of *M. esculenta* polysaccharides into the diet of mice significantly increase the beneficial bacterial density and SCFAs in mice (47, 49). The study by Huo et al. showed the polysaccharides isolated from wild morels had prebiotic effects (54). The polysaccharides with a high molecular weight of 3,947 kDa notably enhanced the relative abundance of *Bacteroidetes, Ruminococcaceae, Erysipelotrichaceae*, and *Lachnospiraceae* in the mice gut. Those microorganisms helped with the digestion of polysaccharides and facilitated the production of SCFAs (87). Rehman et al. also found that *M. esculenta* polysaccharides modulated the gut microbiota in the high-fat-diet (HFD) induced gut dysfunction in obese BALB/c mice (63). The supplementation of both 200 and 400 mg/kg of *M. esculenta* polysaccharides for 12 weeks successfully recovered the diversity of bacteria, decreased the abundance of *Firmicutes* and enhanced the abundance of *Bacteroidetes*, and reduced the risk of obesity and metabolic disorders. Furthermore, at the genus level, the abundance of beneficial bacteria *Lactobacillus* was notably enhanced, indicating an upregulation on the SCFA production. *M. esculenta* polysaccharides after intestinal fermentation significantly inhibited the activities of α-amylase and α-glucosidase, the two important enzymes in carbohydrates digestion, which further helped with diabetic symptoms and gut microbiome regulation (88).

The gut protective effects of *M. esculenta* and *M. importuna* polysaccharides were examined in the streptozotocin-induced type 2 diabetic mice model (64, 89). An improvement in the abundance and diversity of beneficial bacteria were also overserved in the groups treated with 200, 400, and 600 mg/kg of *M. esculenta* polysaccharides (64) and 400 mg/kg of *M. importuna* polysaccharides (89). The situations of both *Firmicutes* and *Actinobacteria* were significantly recovered by *M. esculenta* polysaccharides (63, 64). At the genus level, *Lactobacillus* was improved to a range of 59.1–64.1% compared to 32.1% in the diabetic model. Moreover, the histopathological analysis revealed that *M. esculenta* polysaccharides treatment increased the intestinal permeability *via* upregulating the expressions of colon tight junction proteins such as ZO-1, occludin and claudin-1, and decreased endotoxemia by lowering LPS levels in the diabetic mice (64). Comparatively, *M. importuna* polysaccharides increased the abundance of *Akkermansia*, Blautia, *Dubosiella*, and Lachnospiraceae, and decreased the abundance of Helicobacteraceae, which in turn regulated hepatic metabolism by the gut-liver axis (89).

The flavonoid composition in morels also contributes to its beneficial effects on gut health preservation (90). Xu et al. found flavones from *M. importuna* protected against intestine barrier injury caused by dextran sulfate sodium in mice. The possible mechanisms associated with the elevated diversity and richness of gut microbiota and inhibition of the TLR4/NF-κB signaling pathway (90).

Interestingly, the gut microbiota compositions have mutual connections with the host immune systems and metabolic functions, thus found to be correlated with the development of obesity and diabetes (76). The major mechanism of action of morel polysaccharides lies in the improved diversity and abundance of SCFA-beneficial bacteria. Further investigation on how morel polysaccharides and other bioactive compounds affect their correlations is still needed to help understand their underlying mechanisms.

### 6.5. Anti-cancer activities

Cancer is the leading cause of mortality in most developed countries (91). Although the cancer rate decreased gradually in the US, it remains the biggest puzzle threatening public health. Recent data showed that the most common cancer is lung cancer in China and breast cancer in the US (92). Recently, there has been an increasing number of studies focusing on the anti-cancer activities of morels and their mechanisms of action.

The anti-cancer activities of morels have been observed in colon cancer cell lines. A most recent study investigated the anti-carcinogenic abilities of three morel species- *M. conica, M. esculenta*, and *M. delicosa* from Pakistan (72). Methanolic and ethanolic extracts of the three species of morels showed different anti-proliferative activities toward the SW-480 colon cancer cell. The bioactive compounds that possess the anti-tumor effects in the extracts were mainly amino acids, fatty acids, sterols, flavonoids and phenolic acids. Generally, the methanolic extract of *M. esculenta* showed a better performance in inhibiting the colon cancer growth compared to *M. conica*. The reason could be explained by their different extract compositions. For instance, flavonoid apigenin-7-O-glucoside was found to be presented in *M. conica* to support its anti-tumor activity, and Oleamide was only identified in *M. esculenta*. The methylene chloride extract of *M. esculenta* also demonstrated high antioxidant activity in the HT-29 colon cancer cell (60). The extract containing rich fungal sterols and trilinoleins significantly inhibited the NF-κB which was overactive in the cancer cells, indicating that *M. esculenta* might have potential positive effects on treating colon cancer. In another study, *M. esculenta* polysaccharides with a molecular weight of 81,835 kDa extracted by pulsed electric field inhibited the proliferation of human colon cancer HT-29 cells in a dose and time-dependent manner in the 48 h treatment (65).

MeOH extracts from *M. esculenta* fruiting body were reported to exhibit anti-cancer ability in the human lung adenocarcinoma cells (42). Eight compounds including three fatty acids and five sterols were identified in the MeOH extract. Among the isolated compounds, they found that the derivatives of octadecanoic acid and ergosterol were the main constituents responsible for the ability to induce apoptosis. Other than morels, octadecanoic acid, and ergosterols from other mushrooms have been proven to exhibit anti-tumor abilities in many previous studies (59, 93). They also examined the associated cell mechanisms and found the anti-cancer effects were independent of the tumor-suppressor molecules p53.

DOX (Doxorubicin) is a type of chemotherapy medication called anthracycline antibiotic that inhibits the proliferation of cancer cells (94). Although it remains one of the most cost-effective and active wide spectrum drugs in chemotherapy, its applications are still impeded by the side effects such as high cardiotoxicity and pro-oxidant activity. The polysaccharides from soybean residue fermented *M. esculenta* decreased the oxidative stress in RAW 264.7 macrophages and protected against DOX-induced apoptosis (95). Besides, the polysaccharides MP-1, MP-3, MP-4 induced apoptosis in the liver cancer HepG2 cells and cervical cancer HeLa cells by cell cycle arrest at G0/G1 phase (95).

The anti-tumor effects of the ethanolic extract of *M. esculenta* mycelium were also verified in the animal model (67, 68). The extracts (250, 500, and 1,000 mg/kg body weight) were administered orally for 10 days after the mice were transplanted with tumors. It was found that 1,000 mg/kg of the extract significantly increased the lifespan of tested mice by 55%. Besides, the 250, 500, and 1,000 mg/kg extracts successfully reduced the weight of the transplanted tumor by 41.1, 61.7, and 76.9%, respectively, and reduced the volume of the tumor by 47.9, 59.6, and 74.7%, respectively. The polysaccharide MIPB70-1 from *M. importuna* fruiting body enhanced the anti-tumor activity of DOX, inhibited the growth of breast tumor in BALB/c female mice model, suggesting its potential role as an immune booster (68).

Overall, most cancer studies of morels have been targeting on the colon cancer (60, 65, 72), lung cancer (42), liver cancer (95), cervical cancer (95), and breast cancer (68). The mechanisms include directly inducing apoptosis of tumor cells, enhancing the immune function, and facilitated chemotherapy. These findings collectively supported that the fruiting bodies and mycelia of morels could be potential functional food ingredients or drugs for the prevention and treatment of cancer. However, more animal and clinic trials are still necessary to validate the function and the optimal doses for the morel extracts.

## 7. Conclusion and future direction

Morels are edible mushrooms with excellent nutritional and economic values. Various morel species such as *M. eximia, M. importuna*, and *M. sextelata*, are cultivated in China, while *M. conica* and *M. esculenta* are most commonly found in the US. *M. esculenta* is the morel species that has been most studied. The life cycle of morels is complicated and lacks thorough understanding, thus the large-scare artificial morel cultivation remains a challenge. This relatively low yield and high market price of morels causes their consumption less prevalent than other common edible mushrooms, such as shiitake, oyster mushrooms and white mushrooms. Studies about increasing the spawn quality, optimizing the exogenous nutrition to support morel growth and reproduction, advanced indoor cultivation technology are still ongoing to conquer the challenge.

Morels are rich in proteins, fibers, necessary vitamins, minerals, and PUFAs. Recently, a trend toward increasing plant-based foods and reducing meat consumption is growing, and alternative meat is becoming popular (96, 97). Morels have umami taste, meaty flavor and spongy texture. These unique characteristics make morels distinguished substitutes for meat in vegetarian and flexitarian diet. In a typical meat analog recipe, protein (10–25%) is the second most ingredient following water (50–80%) (98). Protein from soy, wheat, legume has been frequently used in meat analogs, but industry is still searching for other protein-rich ingredients. Since the crude protein content of fermented mycelia of *M. esculenta* reached 39.4% and their pleasant flavor, morel mycelia from submerged fermentation may became a novel protein source and a potential alternative to animal protein (17, 99). There are two examples of food brands, Quorn and Meati, who have successfully used biomass fermentation of filamentous fungi as the base for meat analogs and launched to market (100). With more attention to submerged fermentation technology including species determination, medium optimization, bioreactor design, and protein and amino acids identification, morel mycelia would have a great opportunity in area of meat alternative.

The bioactive constituents in morels vary among species and origins, and they are mainly composed of polysaccharides, phenolics, tocopherols, ergosterols etc. These bioactive compounds in morels greatly contribute to their health benefits including anti-oxidative, anti-inflammatory, immunoregulative, gut protective, and anti-cancer abilities. The major underlying cell mechanisms of morels explaining their health benefits involved the regulation of TLR4-NF-κB and MAPK signaling. Up to now, most studies have focused on the polysaccharides and phenolics of morel fruiting bodies, and these extracts can be developed and further applied into dietary supplement. More studies about absorption, metabolism, distribution, and excretion of morel extracts in animals should be conducted and safety assessment is necessary.

Considering the high value of morel fruiting bodies and the mycelia, the future directions of morel studies can be extended but not limited to developing new technologies toward the cultivation and fermentation of mycelia, exploring the health benefits of various morel species and mycelia, and applying morel extracts into the development of novel functional foods. Besides, further research on the molecular mechanisms of morel extracts and other minor bioactive compounds are still desired to understand this valuable mushroom and broaden its applications.

## Author contributions

YL: writing—original draft and visualization. HC: writing—review and editing. XZ: writing—sections, review and editing, funding acquisition, and conceptualization. All authors contributed to the article and approved the submitted version.
